# COVID-19 effects on national health system response to a local epidemic: the case of cerebrospinal meningitis outbreak in Ghana

**DOI:** 10.11604/pamj.supp.2020.35.2.23031

**Published:** 2020-05-05

**Authors:** Derrick Mensah, Robert Asampong, Paul Amuna, Martin Amogre Ayanore

**Affiliations:** 1 School of Public Health, University of Health and Allied Sciences, Ho, Ghana

**Keywords:** COVID-19, cerebrospinal meningitis, national health system, Ghana

## To the editors of the Pan African Medical Journal

COVID-19 will go down in history as one pandemic that has tested the preparedness of national health systems to combat health emergencies globally. Globally, no country that has since recorded cases of COVID-19 can argue that this pandemic has not impacted negatively on their national health systems. As at 19th April, 2020, 09:20 GMT, Africa´s case fatality numbers have passed the 10,000 mark [1]. In Ghana, the number of confirmed cases had passed 1000 [2] with ten out of the sixteen administrative regions in the country reporting cases [3]. In line with WHO recommendations and prompt national priority-setting targets to mitigate the effects of the pandemic, the government of Ghana set-up a national response team to immediately put in place preparedness plans and coordinate all activities in a bid to control the effects of COVID-19 on the Ghanaian economy.

Across Africa, while there is no argument that COVID-19 is a high priority public health issue that national governments have to battle with and find an effective way to contain its spread, local epidemics appear to be happening with seemly low inertia from the same national health systems. In Ghana, there is an outbreak of cerebrospinal meningitis (CSM), with growing public health concern about the increasing number of cases. The Ghana Health Service (GHS) on 15th April, 2020 reported a total of 409 cases count of CSM in five out of the sixteen regions in Ghana [4]. The GHS reported over 40 deaths by the second week of April 2020, far surpassing the case fatality of 9 deaths for COVID-19 within the same time-frame. While the magnitude of the CSM outbreak is severe and unprecedented in a decade, the slow pace of national response to the CSM epidemic, largely due to the fact that the national health team is overstretched in managing COVID-19 is a cause for worry.

The cause of the current CSM outbreak is a new strain of bacteria known as the Neisseria meningitidis serotype X and Streptococcus pneumonia which has an average case fatality of 40% [5]. Unfortunately, a licenced vaccine against the new serotype is non-existent, making it even much worse in the midst of a global pandemic. According to the WHO, about 10-15% of patients diagnosed with CSM will die if not treated [6]. The current number of deaths implies that CSM has killed about 4 times that of COVID-19 in the country. The reason why Ghana has recorded such a high number of deaths from CSM is because much attention has been shifted to fighting COVID-19. Undoubtably, environmental precipitated factors, poor built-environment including housing and rising temperatures have been the causes of CSM outbreaks in Ghana over the years. Sadly, the CSM outbreak became public knowledge after a communication from health authorities after over 30 people lost their lives [7].

The communication from state health authorities was intended to draw public attention to the detection and deaths recorded from the outbreak and to call for public support in adhering to health safety measures to contain the outbreak. A Ghanaian lawmaker calls the slow pace of public health actions to contain the CSM outbreak a “loud silence and lack of expeditious action” [7] as increased calls from the public grow for resources to be committed to tackle the outbreak. Ghana´s Ministry of Health (MoH) and the GHS are beginning to act, recognizing that careful planning in resource allocation and the deployment of frontline health workers in the midst of COVID-19 is vital to contain the CSM outbreak. The high case fatalities from the CSM outbreak and the daily rising case counts of COVID-19 in Ghana show a clear need for health systems strengthening that offer a continuum of care, both for persons directly affected by these health crises and the general population who may need other forms of health care support.

It is vital that national health responses during emergencies do not neglect other pressing needs of the health care system, as this can further bridge inequalities in access for primary health care services. It is also important that health authorities apply a broader health system thinking approach in crises such as these to act continuity for the health care system, prioritizing building resilience for the health system and for future health emergencies. Also, the health system must leverage resources across epidemic control teams to avoid waste and ensure task shifting roles are activated.

## Conclusion

A CSM outbreak alongside COVID-19 in Ghana means national health authorities have to deal with a double health emergency. At the level of health policy, preparedness plans and their implementation strategies must not revolve around single national response teams, as this has a potential to delay health system response to either CSM and COVID-19 currently affecting livelihoods, health and well-being and the Ghanaian economy. A strong policy commitment to fund CSM research by academics and research institutes is needed in Ghana. Long-term health system priorities based on empirical research on the drivers of CSM and what health system interventions can best address CSM is vital for long term health system control and mitigation of the disease.

## Competing interests

The authors declare no competing interests.
